# Gimap5 Inhibits Lung Cancer Growth by Interacting With M6PR

**DOI:** 10.3389/fonc.2021.699847

**Published:** 2021-09-15

**Authors:** Pei Dai, Zhongxiang Tang, Pinglang Ruan, Ousman Bajinka, Dan Liu, Yurong Tan

**Affiliations:** ^1^Department of Medical Microbiology, Xiangya School of Medicine, Central South University, Changsha, China; ^2^China-Africa Research Centre of Infectious Diseases, School of Basic Medical Sciences, Central South University, Changsha, China

**Keywords:** lung cancer, GIMAP5, M6PR, PADI4, prognosis, potential biomarker

## Abstract

**Objective:**

Several studies have demonstrated the impacts of GTPases of immunity-associated proteins (GIMAPs) on malignant cells. However, the mechanisms through which Gimap5 regulates lung cancer cells are yet to be thoroughly investigated in the literature. Our study aimed to investigate the function of Gimap5 in the development of lung cancer.

**Methods:**

The expression levels of the GIMAP family were analyzed in lung cancer patients of various cancer databases and lung cancer cell lines. After the survival rates of the cells were analyzed, we constructed Gimap5 over-expressed lung cancer cell lines and assessed the effects of Gimap5 on cell migration, cell invasion, cell proliferation and the epithelial-mesenchymal transition (EMT). We later screened the interacting proteins of Gimap5 using Co-IP combined with mass spectrometry and then analyzed the expression and distribution of M6PR, including its impacts on protein-arginine deiminase type-4 (PADI4).

**Results:**

Findings indicated that GIMAP family expression decreased significantly in lung cancer cell lines. We also noticed that the downregulation of the GIMAP family was related to the poor prognosis of lung cancer patients. Our experimental results showed that Gimap5 could inhibit the migration, invasion, proliferation and EMT of lung cancer cell lines. Moreover, we found that Gimap5 promoted the transport of M6PR from the cytoplasm to the cell membrane, thereby inhibiting the enhancement of EMT-related PADI4.

**Conclusion:**

Our research suggested that Gimap5 could inhibit the growth of lung cancer by interacting with M6PR and that it could be a potential biomarker for the diagnosis and prognosis of lung cancer.

## Introduction

Lung cancer is one of the leading causes of cancer-related deaths globally. With over 1.8 million new cases each year, this malignancy accounts for 11.6% of all cancers worldwide (1–3). Lung cancer has two main histological subtypes: non-small cell lung cancer (NSCLC) and small cell lung cancer (SCLC) (4). NSCLC is a common form of lung cancer. It accounts for more than 80% of the total incidence of lung cancer, and its 5-year overall survival rate is relatively poor (5). Lung cancer is prone to metastasis, and at least one third of patients develop brain metasta (6).This cancer has been treated with radiation therapy, chemotherapy, and surgery. However, the health conditions of patients treated with these clinical methods have not improved considerably (7, 8). Hence, it is crucial to develop formidable treatment methods that can improve the 5-year overall survival rate of patients with this cancer.

The GTPases of immunity-associated protein (GIMAP) family is a large group of GTP enzyme hydrolases that are involved in various cellular pathways, such as protein synthesis, signal transduction, and vesicle transportation (9, 10). This family consists of seven members in human: GIMAP1, GIMAP2, GIMAP4, GIMAP5, GIMAP6, GIMAP7, and GIMAP8. The GTPase domain of the GIMAP protein is relatively small, with molecular sizes ranging from 34 to 38 kDa. In experimental models and studies on human pathology, specific members have been shown to be involved in lymphocyte development and to be associated with inflammatory and autoimmune diseases. Located in the endoplasmic reticulum, lysosome, Golgi body and mitochondria, Gimap5 is highly expressed in the lymph nodes and spleen. While T lymphocytes (i.e., CD4 and CD8 positive T cells) and monocytes were highly expressed, the expression of B lymphocytes was very low (11). Studies have found that Gimap5 could influence the survival of T cells and that polymorphisms in human Gimap5 genes are related to autoimmune diseases (12, 13). Besides, the absence of Gimap5 promotes the development of pathogenic T cells and airway allergic diseases with an increase in the Th17/Th2 proportion (14). There are few studies on Giamp5 in tumors. Therefore, the main purpose of this study is to explore the role of Gimap5 in the occurrence and development of lung cancer.

M6PR is a membrane integrator glycoprotein that can be divided into two groups based on the divalent ion that relies on the M6PR activity. The first group of the binding activity is CI-M6PR, which does not depend on divalent ions (15). The second group is CD-M6PR, which depends on divalent ions (16). Often referred to as IGFII/M6P receptors, M6PR is used as a sorter and deliverer of lysosomal enzymes. Lysosomal enzymes can bind to the extracellular region of M6PR through its M6P marker, and they are sorted and delivered through the receptor-mediated transport. Morgan (17) found that CI-M6PR was a receptor for insulin-like growth factor II (IGFII). The lysosome sorting through CI-M6PR is usually more effective than CD-M6PR. This indicates that CI-M6PR in mammalian cells is the main lysosomal targeting receptor (18). However, M6PR can bind to other targets that affect cell proliferation, migration, and invasion, including IGF-II, growth factor β, the urokinase-type plasminogen activator receptor, and plasminogen (9, 15, 19–21). Therefore, the inactivation of the M6PR gene is highly correlated with the tumor (22), and this gene acts as a tumor suppressor or inhibits tumor growth (23). In one study, the development of breast tumors was found to be impaired in transgenic mice with M6PR receptor overexpression (24). M6PR overexpression was also discovered to reduce cancer cell growth *in vitro* and *in vivo*. In some cases, it simultaneously promoted cell death (25).

Based on previous findings, we focused on the impacts of Gimap5 on the occurrence and development of lung cancer. We hypothesized that a relationship existed between lung cancer and Gimap5. As well as informing scholarship on lung cancer, this research could also be relevant in providing insights into the biomarkers that could improve the diagnosis and treatment of lung cancer.

## Material and Methods

### TCGA Data Analysis

The Cancer Genome Atlas (TCGA) database analysis tool (http://gepia.cancer-pku.cn/) was used to analyze the expression of GIMAP family genes in lung squamous cell carcinoma (LUSC) and lung adenocarcinoma (LUAD) (LUAD tumor sample, 483; normal sample, 347; LUSC tumor sample, 486; normal sample, 338).

### GEO Microarray Data

Five mRNA gene expression profiles (GSE19804, GSE33532, GSE27262, GSE101929 and GSE21933) were downloaded from the National Center for Biotechnology Information Gene Expression Omnibus database (https://www.ncbi.nlm.nih.gov/geo/). GSE101929 included 33 tumor samples and 33 paired non-tumor samples, while GSE33532 consisted of 80 tumor samples and 20 corresponding normal samples. GSE27262 was comprised of 25 tumor samples and 25 normal samples. GSE19084 consisted of 60 tumor samples and 60 normal samples, while GSE21933 consisted of 21 tumor samples and 21 normal samples. The raw microarray data files (CEL files) of the five datasets were downloaded from the GEO database. The background correction and quantile normalization were performed using the Robust Multichip Average (RMA) algorithm of the R package Affy. Subsequently, the linear models for the microarray data (limma) package in R were used to calculate the probability of probes being differentially expressed between cases and controls. The fold change (FC) and its logarithm value (log FC) were also determined. The corrected p-values < 0.05 and absolute∣log2 fold change∣> 1 were used to identify mRNAs that were differentially expressed significantly.

### Survival Analysis of the GIMAP Family

We analyzed the overall survival rate for lung cancer using the online survival analysis tool (http://gepia.cancer-pku.cn/ and http://kmplot.com/analysis/)

### Cell Culture and Transfection

The human normal lung epithelial cell Beas-2b, the lung cancer cell line A549, PC9, and 1299 were frozen and retained by the Department of Microbiology, Xiangya School of Medicine, Central South University (Hunan, China). The cells were cultured under sterile conditions and maintained in Dulbecco’s Modified Eagle’s Medium (DMEM) or the RPMI 1640 medium supplemented with 10% fetal bovine serum (FBS, Gibco, USA) and 1% Penicillin-Streptomycin-Amphotericin B Solution. They were then incubated at 37°C in a 5% CO_2_ atmosphere.

Furthermore, pcDNA3.1-Gimap5/3×Flag-EGFP and pcDNA3.1-Control were constructed by Genscript Company (Beijing, China), and siM6PR plasmids were synthesized by Sangon Biotech (Shanghai, China). The NEOFECT™DNA transfection reagent was purchased from NEOFECT Company (Beijing, China). The Gimap5 over-expressed cell lines (Beas-2b, A549, PC9 and 1299 cell lines) were constructed using NEOFECT according to the manufacturer’s protocol. The siRNA sequence used to interfere with M6PR included F: 5 ‘- GCUCUAGUGAAGAGGCUGAAATT- 3’ and R: 5 ‘- UUUCAGCCUCUUCACUAGAGCTT - 3’. The primer sequences used in this study were listed in **Table 1**.

**Table 1:** The primer sequences used in the study.

Gene	primer sequence (5’-3’)
GAPDH	F: 5’-GCACCGTCAAGGCTGAGAAC-3’
	R:5’-TGGGAAGACGCCAGTGGA-3’
Gimap5	F:5’- CCCTCCATCTTTGAGTCACAGG -3’
	R:5’- CTGTGTCCTGAGCAGTGAAACG -3’
M6PR	F:5’- TTGAGTGGCGAACGCAGTATGC -3’
	R:5’- CAGTGATGGCTTCCCAGTTGTC -3’
E-cadherin	F:5’- GCCTCCTGAAAAGAGAGTGGAAG -3’
	R:5’- TGGCAGTGTCTCTCCAAATCCG -3’
N-cadherin	F:5’- CCTCCAGAGTTTACTGCCATGAC -3’
	R:5’- GTAGGATCTCCGCCACTGATTC -3’
PADI4	F:5’- GCACAACATGGACTTCTACGTGG -3’
	R:5’- CACGCTGTCTTGGAACACCACA -3’
Snail	F:5’- TGCCCTCAAGATGCACATCCGA -3’
	R:5’- GGGACAGGAGAAGGGCTTCTC -3’
Slug	F:5’- ATCTGCGGCAAGGCGTTTTCCA -3’
	R:5’- GAGCCCTCAGATTTGACCTGTC -3’
ZO-1	F:5’- GTCCAGAATCTCGGAAAAGTGCC -3’
	R:5’- CTTTCAGCGCACCATACCAACC -3’
Vimentin	F:5’- AGGCAAAGCAGGAGTCCACTGA -3’
	R:5’- ATCTGGCGTTCCAGGGACTCAT -3’
Twist	F:5’- GCCAGGTACATCGACTTCCTCT -3’
	R:5’- TCCATCCTCCAGACCGAGAAGG -3’
Caspase3	F:5’- GGAAGCGAATCAATGGACTCTGG-3’
	R:5’- GCATCGACATCTGTACCAGACC -3’
Caspase9	F:5’- GTTTGAGGACCTTCGACCAGCT-3’
	R:5’- CAACGTACCAGGAGCCACTCTT-3’

### Invasion and Migration Assay

For invasion assays, the Matrigel matrix was diluted in serum-free DMEM (1:9 concentration). After that, 50 μl of the diluted Matrigel matrix was coated on the basement membrane surface of the upper chamber. However, no Matrigel matrix was needed for the migration assay. The cells with Gimap5 overexpression were then digested, resuspended (10^5^ cells in 200 μl DMEM without serum) and placed on the upper chamber of the Transwell chamber. The lower chamber was placed in a culture medium (600 μl) containing 20% FBS. The cells were subsequently incubated for 24-36 h at 37°C in an atmosphere containing 5% CO_2_. After the cell suspension in the upper chamber was discarded, the cells on the upper surface were removed with a cotton swab. The cells on the lower surface were then stained with 4% crystal violet. Afterward, they were incubated at room temperature for 15 min and then washed three times with PBS. The invaded and migratory cells were observed and counted under an inverted microscope (Leica, Weitzlar, Germany).

### Cell Proliferation Assessment

For CCK-8 assay, PC9, A549 or 1299 cells with Gimap5 overexpression were digested and cultured in a 96-well plate at a density of 1×10^3^ cells/well. After 24 h of incubation, CCK-8 solution (10 μl) was added to each well and cultured at 37°C for 2 h. The spectrophotometric absorption value of each well at 450 nm was measured using TECAN F50 (Mannedorf, Switzerland). Triplicate was set up in each assay. Besides, independent experiments were carried out at least three times, and the average OD value was calculated.

### Cell Migration Assessment

The cells were seeded in 6-well plates and then cultured. After the cells with Gimap5 overexpression reached 90% confluence, the tip of the sterile micro liquid remover (10 μl) was used to underline the plate and draw a line every 1 cm. Subsequently, the exfoliated cells were washed with sterile PBS. After the cells were cultured for 24 h, photographs were taken by the inverted microscope, and the wound width was measured using Image J software. Triplicate was set up in each assay, and independent experiments were carried out at least three times.

### Flow Cytometry for Annexin V-FITC/PI Labeling

Apoptotic cells labeled with Annexin V-FITC/PI were used for the evaluation of phagocytosis by flow cytometry, as described in Annexin V-FITC/PI Apoptosis Detection Kit (Cat No. 40302, YEASEN, Shanghai, China). Briefly, the pretreated cells were collected and centrifuged at 300g at 4°C for 5min, then precooled and washed twice with PBS. Cells from 1 to 5×10^5^ were collected. PBS was discarded and 100 μl of 1×Binding Buffer was added to resuspend the cells. Add 5 μl Annexin V-FITC and 10 μl PI staining solution and mix gently. After adding 1×Binding buffer, the samples were darkened and kept at room temperature for 10-15 min. The samples were mixed and detected by flow cytometry within 1h. The cells were analyzed and sorted using a BD FACS Aria™ II Cell Sorter (BD Biosciences, Franklin Lakes, NJ, USA). The data obtained were analyzed using FlowJo version 10.6 software (FlowJo, LLC, Ashland, OR, USA).

### Quantitative Real-Time Polymerase Chain Reaction

The total RNA was isolated from the cells using TRIzol Reagent (TaKaRa, Kusatsu, Japan). Afterward, the RNA was subjected to reverse-transcription using the PrimeScript 1st Strand cDNA Synthesis Kit (TaKaRa, Kusatsu, Japan) according to the manufacturer’s instructions. Next, qRT-PCR was performed using the 2 × SYBR Green qPCR Master Mix (Bimake, Houston, USA). The relative mRNA expression levels of Gimap5, M6PR, E-cadherin, N-cadherin, Snail, PADI4, Slug, Caspase3 and Caspase9 were subsequently normalized using GAPDH. The data collected were presented as the mean value ± S.D (26).

### Western Blotting Assessment

The total proteins were extracted from the cultured cells in the RIPA buffer containing protease inhibitors. After all the cell lysates were separated by SDS-PAGE, they were transferred to the polyvinylidene fluoride membrane (PVDF). The PVDF membrane was blocked by 5% skim milk at room temperature for 1 h. The membranes were then incubated overnight with the primary antibody at 4°C. The primary antibody included rabbit anti-Gimap5 (14108S, Cell Signaling Technology, Boston, USA, 1:1000), rabbit anti-E-cadherin (bs-1519R, BIOSS, Beijing, China, 1:1000), rabbit anti-N-cadherin (bs-20623R, BIOSS, Beijing, China, 1:1000), rabbit anti-CD-M6PR (BS8108, Bioworlde, Beijing, China, 1:1000), rabbit anti-CI-M6PR (WL02758, Wanleibio, Shenyang, China, 1:500), rabbit anti-PADI4 (BS7314, Bioworlde, Beijing, China, 1:1000) and rabbit anti-GAPDH (BIOSS, Beijing, China, 1:5000). After each membrane was washed with TBST (containing 1% Tween 20) six times for 5 min, all the membranes were incubated with horseradish peroxidase-conjugated secondary antibodies (BIOSS, Beijing, China, 1:5000) at room temperature for 1 h. The membranes were eventually visualized using a Tanon 5200 chemiluminescence image analysis system (Tanon, Shanghai, China).

### Co-Immunoprecipitation and Tandem Mass Spectrometry

The Beas-2b and PC9 cells with Gimap5 overexpression were first lysed with the lysis buffer (50mM Tris HCl, pH7.4, 150mM NaCl, 1mM EDTA, and 1% Triton X-100). The extracted protein samples were then co-immunoprecipitated with rabbit anti-Gimap5 (10 μg) and IgG (BIOSS, Beijing, China), respectively. Rabbit-anti M6PR (10 μg) was subsequently added to perform secondary immunoprecipitation. Afterward, the target proteins and their interacted proteins were co-immunoprecipitated by Protein A-Agarose (SC-2001, SANTA CRUZ, California, USA), and the unbound proteins were eliminated three times using PBS elution. The collected samples were then isolated using SDS-PAGE 12% bis-Tris protein gel. Next, they were transferred to the PVDF membrane and tested with the goat-anti-rabbit secondary antibody (BIOSS, Beijing, China). Finally, the remaining samples were used for tandem mass spectrometry analysis, which was performed by Jingjie Biotechnology (PTM.BIO Lab, Zhejiang, China).

### Structural Modeling and Molecular Docking of Gimap5 and M6PR

The 3D model of GIMAP5 was performed with the SWISS-Model online tool. 3D Shapes of M6PR was also downloaded from the Protein Data Bank (PDB ID:3CY4; www.rcsb.org). Following that, the protonization and energy minimization was determined. The energy approximation of docking structures was later simulated with the London-DG scoring function. The ligand interaction tool of Autodock Vina software was subsequently used to detect the connection of protein-small molecules. The Chimera and Pymol tools of UCSF were finally employed to generate the PDB model of the target small molecule 3D structure (27).

### Indirect Immunofluorescence and Co-Localization Analysis

PC9 cells with Gimap5 overexpression were added to the Fisher Coverglass. After the cells adhered to the cover glass, the cells were fixed with 4% ice-cold paraformaldehyde and treated with 0.1% Triton X-100. After that, the cells were blocked with 1% BSA at room temperature for 30 min. Next, the rabbit anti-CI-M6PR antibody (1:200) was used for staining, and the staining was performed overnight at 4°C. Mouse anti-Gimap5 (SC-377307, SANTA CRUZ, California, USA, 1:500) was added for co-location staining. The next day, the TRITC goat anti-rabbit antibody and the FITC goat anti-mouse fluorescent antibody (Zen-Bioscience, Chengdu, China, 1:300) were added to the cover glass and incubated in the dark at room temperature for 1 h. After the cells were washed in PBST (including 1% Tween 20) five times for 5 min and stained with DAPI for 10 min, the co-localization of Gimap5 and M6PR were observed under the confocal laser scanning microscope.

### Statistical Analysis

GraphPad Prism was used to perform statistical analysis. All the collected data were from at least three independent experiments, and they were presented as the mean ± standard deviation (SD). The Student t-test and the two-way ANOVA were used to compare the statistical differences between two groups and between multiple groups, respectively and least significant difference (LSD) was used as a post-hoc test. Data with p-values < 0.05 were assumed to be statistically significant.

## Results

### GIMAP Family Gene Expression Was Downregulated in Lung Cancer

**Figure 1:**
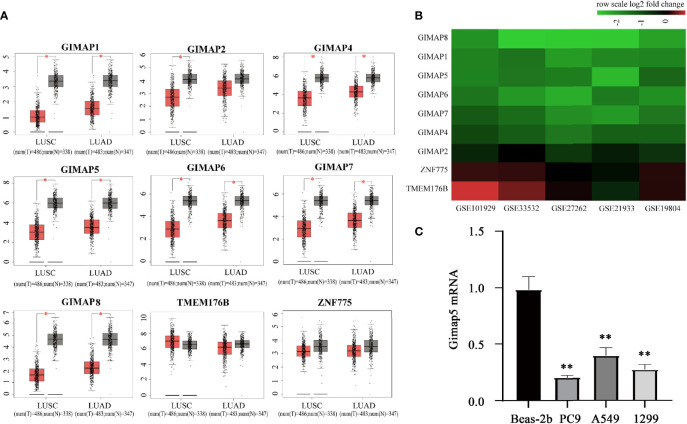
GIMAP family expression were downregulated in lung cancer. **(A)** GEPIA boxed plots of GIMAP family in human LUSC, LUAD and normal lungs from TCGA. Transverse axis represents different lung cancer types, LUSC and LUAD and longitudinal axis represents a multiple of the difference in GIMAP gene expression between cancer tissue and normal tissue, and red for lung cancer tissue, gray for normal tissue. *p < 0.01 (LUSC: tumor = 486, normal = 338; LUAD: tumor = 483, normal = 347). **(B)** The GIMAP family gene was significantly down-regulated in five data sets (abscissa axis), but there were no changes in TMEM176B and ZNF775 adjacent to the family genes (axis of ordinates), red represents up-regulation, green represents down-regulation. The darker the color, the more obvious the difference in expression. **(C)** Gimap5 expression in various lung cancer cell line or human bronchial epithelial cells (n = 3). **p < 0.01 *vs*. Beas-2b cells.

We explored the expression profile of the GIMAP family genes in LUSC and LUAD by using the GEPIA database (http://gepia.cancer-pku.cn/) (28). The GEPIA box plots of GIMAP expression levels showed that while the expression of seven genes (GIMAP1, GIMAP2, GIMAP4, GIMAP5, GIMAP6, GIMAP7 and GIMAP8) was low in LUAD and LUSC, the downregulation of GIMAP2 was not significant compared to the other six genes in LUAD (**Figure 1A**). However, the adjacent gene ZNF775 and TMEM176B (GIMAP family genes) were not altered.

Furthermore, we confirmed the downregulation of GIMAPs in the TCGA database. Five NSCLC gene expression profiles (GSE19804, GSE33532, GSE27262, GSE101929, and GSE21933) were analyzed to confirm the expression levels of the GIMAP family genes during tumorigenesis. The mRNA expression levels in the GIMAP family were found to be significantly lower in NSCLC than in normal lung tissues. In addition, the amplitude of GIMAP2 downregulation was significantly weaker than that of the other GIMAP genes. It was also found that chromosome adjacent genes ZNF775 and TMEM176B did not exhibit any significant changes (**Figure 1B**). The expression of GIMAP5 in various lung cancer cell lines was detected using qRT-PCR, such as PC9, A549 and 1299 and normal human bronchial epithelial cells (Beas-2b). Experimental results revealed that the expression levels of GIMAP5 in all lung cancer cell lines were lower than those in Beas-2b cells (**Figure 1C**). In sum, these data indicated that GIMAP5 was downregulated in lung cancer.

### Downregulation of GIMAP Family Genes Was Associated With Poor Prognosis

**Figure 2:**
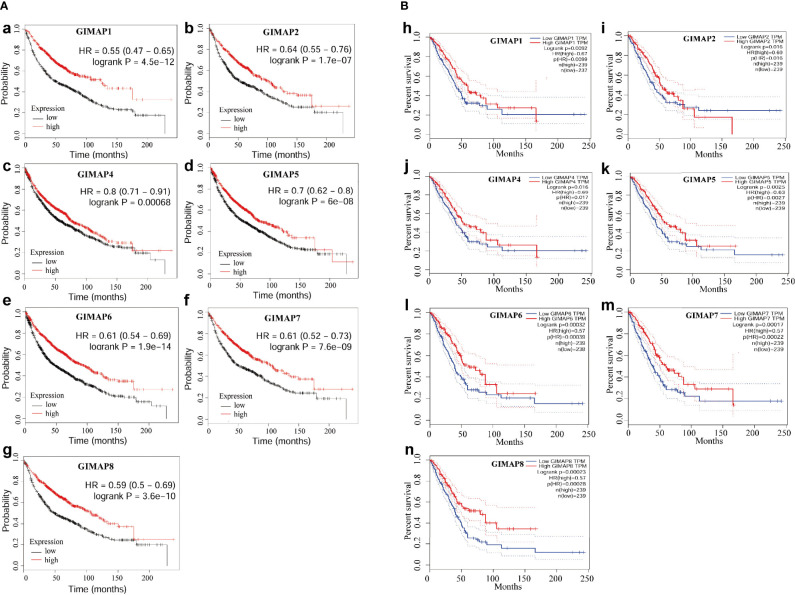
Down-regulation of GIMAP family was associated with poor prognosis of lung cancer. **(A)** Overall survival analysis of GIMAP family in lung cancer patients from TCGA database (a-g), red lines represent GIMAP higher expression, the higher survival probability and black lines represent the lower GIMAP expression, the lower the survival probability. **(B)** Prognostic value of GIMAP family in lung cancer patients from GEO database. Log-rank test was performed to evaluate the survival differences between the two curves (h-n), red lines represent GIMAP higher expression, the higher survival and blue lines represent the lower GIMAP expression, the lower the survival rate.

The survival analysis of the GIMAP family was performed, and the results showed that high levels of GIMAP1, GIMAP2, GIMAP4, GIMAP5, GIMAP, GIMAP7 and GIMAP8 mRNA expression levels were associated with better overall survival of patients with lung cancer (**Figure 2A**). The overall survival analysis of the GIMAP family was also performed using the Kaplan–Meier plotter online tool (http://kmplot.com/analysis/). The results showed that high levels of all members of the GIMAP family were associated with better overall survival of patients with lung cancer (**Figure 2B**).

### Gimap5 Upregulation Inhibited the Invasion, Migration and Proliferation of Lung Cancer Cells and Promotes Cell Apoptosis

**Figure 3:**
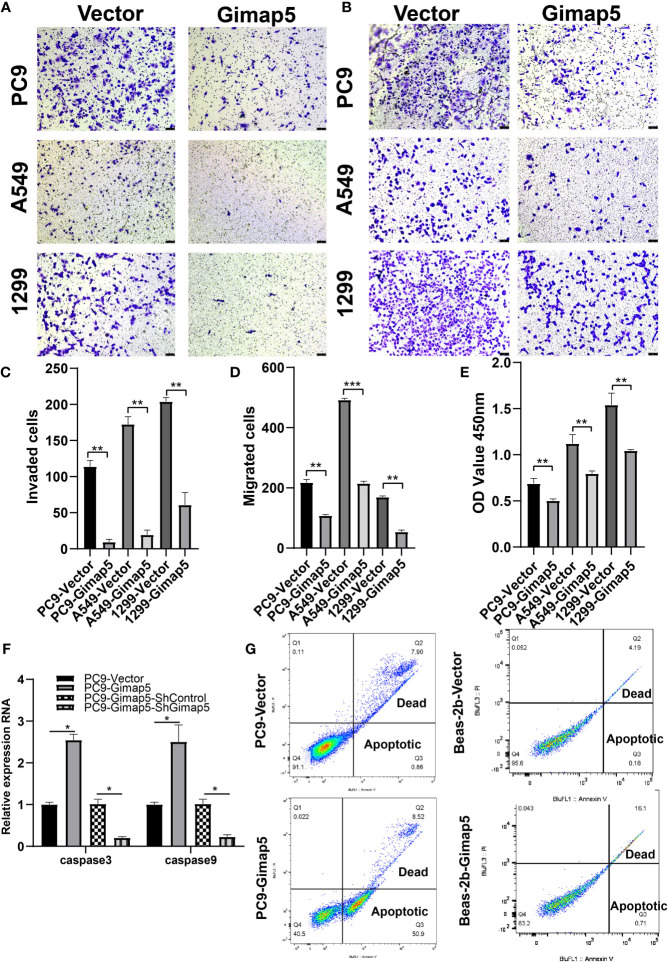
Gimap5 inhibited migration, invasion and proliferation and promoted apoptosis of lung cancer cells (n = 3). **(A)** Gimap5 overexpression inhibited the invasion of all three cancer cell lines using Transwell assay. **(B)** Gimap5 overexpression inhibited the migration of all three cancer cell lines using Transwell assay(magnification, ×100). **(C, D)** The numbers of invading and metastasized cells were measured by Image J and represented by bar graph. **(E)** Gimap5 overexpression inhibited the proliferation of all three cancer cell lines using CCK-8 assay. The data were expressed as mean ± SEM (**p < 0.01 and ***p < 0.001 *vs*. empty vector) of the three independent experiments. **(F)** Gimap5 overexpression promoted apoptosis. After overexpression of Gimap5, the mRNA expression levels of caspase3 and caspase9 were up-regulated (*p < 0.05 vs. empty vector). After knockout of Gimap5, the mRNA expression levels of caspase3 and caspase9 were down-regulated (*p < 0.05 vs. shControl). **(G)** Flow cytometry showed that overexpression of Gimap5 significantly increased cell apoptosis in PC9 cells, however, there was no significant change in Beas-2b cells. In the scatter plot of the bivariate flow cytometer, the lower left quadrant shows living cells, which is (FITC^-^/PI^-^); The upper right quadrant is non-living cells, i.e. necrotic cells, and is (FITC^+^/PI^+^); The lower right quadrant is apoptotic cells, showing (FITC^+^/PI^-^).

To explore the biological function of Gimap5 in lung cancer progression, we constructed Gimap5-overexpressed cell lines for PC9, A549 and 1299 cells. This was because their expression levels in lung cancer were extremely low. After assessing the effect of Gimap5 on lung cancer invasion and migration *in vitro* using Transwell analysis, we found that the invasion abilities of cells with Gimap5 overexpression were significantly downregulated and that they decreased by 60% compared with the control group (**Figures 3A, C**). We also noticed that the migration abilities of the cells were reduced by 45% (**Figures 3B, D**). Besides, we found that Gimap5 overexpression inhibited cell proliferation but promoted the apoptosis of all three cancer cell lines (**Figures 3E–G**).

### Gimap5 Upregulation Inhibited Migration and EMT

**Figure 4:**
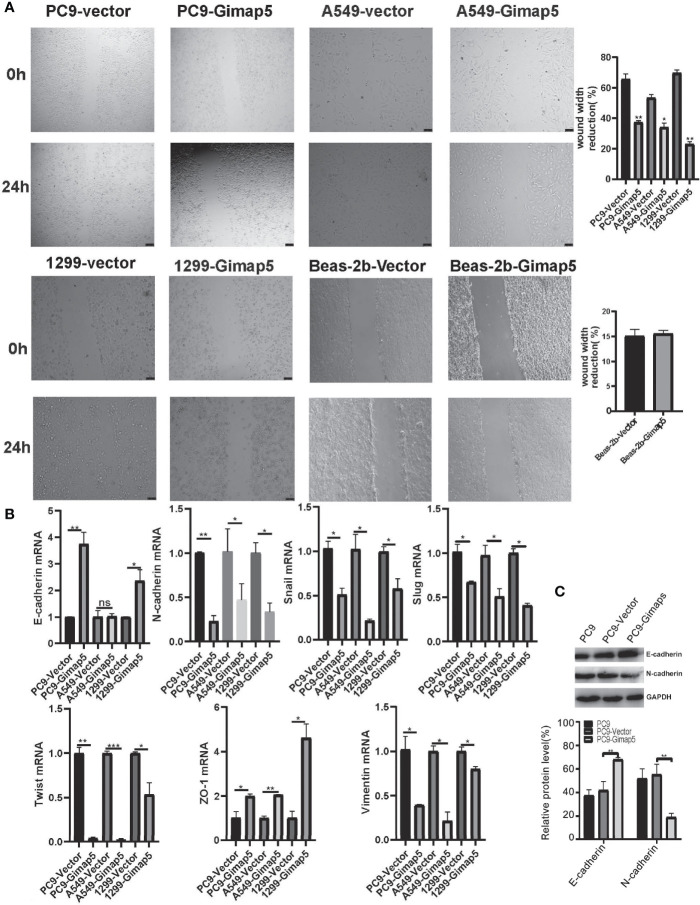
Gimap5 upregulation inhibited migration and EMT (n = 3). **(A)** Migration speed of PC9, A549, 1299 and Beas-2b cells (magnification, ×100). **(B)** The mRNA expression levels of EMT-related biomarker were assayed using qRT-PCR, and the data were expressed as mean ± SEM of the three independent experiments. **(C)** Western blot was used to detect the expression of E-cadherin and N-cadherin proteins in PC9 cells. After the image scanning gray analysis, the internal reference. GAPDH correction was used to obtain the ratio of each group. (ns means no significance, *p < 0.05 ,**p < 0.01 and ***p < 0.001 vs. empty vector) .

In the scratch migration experiment, we found that Gimap5 overexpression significantly decreased the proliferation and migration abilities of lung cancer cell lines compared with those in the control group (**Figure 4A**). Epithelial-mesenchymal transformation (EMT) is one of the most important mechanisms involved in tumor invasion and metastasis (29). To further verify the ability of Gimap5 to regulate lung cancer metastasis, we studied the effect of Gimap5 on the expression levels of EMT biomarkers (E-cadherin, N-cadherin, Snail, Slug, Twist, ZO-1, Vimentin) in PC9, A549 and 1299 cells. We found that in the Gimap5 overexpressed cell lines, E-cadherin and ZO-1 were upregulated, while Snail, Slug, Twist, Vimentin and N-cadherin were downregulated (**Figure 4B**). The results of Western blotalso showed that E-cadherin expression was up-regulated and N-cadherin expression was down-regulated (**Figure 4C**). Meanwhile, we also investigated the effect of Gimap5 overexpression on EMT markers in Beas-2b cells. It was found that Gimap5 overexpression also increased E-cadherin and significantly decreased Slug, but had little effect on N-cadherin and Snail (**Supplementary Figure 1**). This suggests that Gimap5 overexpression can reduce the tendency of EMT in normal cells.

### Gimap5 Interacted With M6PR

**Figure 5:**
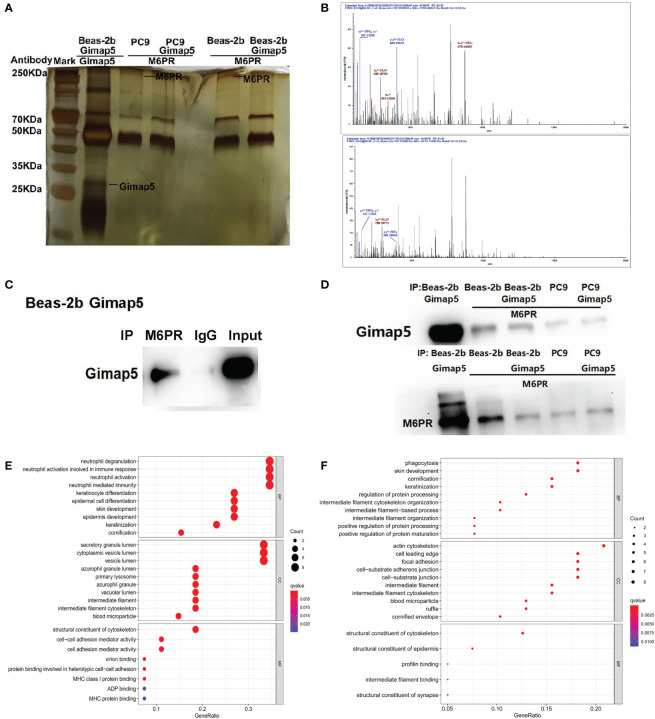
The interacted proteins of Gimap5 were screened using combined Co-IP and LC-MS/MS. **(A)** Silver staining map of Co-IP assay using Gimap5 antibody after transfection of Beas-2b or PC9 cells with Gimap5 overexpressed vector. The first lane represents marker; second lane is Co-IP using Beas-2b cells; the third lane is M6PR antibody to the Gimap5 IP products of the PC9 cells; the fourth lane is M6PR antibody to the Gimap5 IP products of the PC9-Gimap5 cells; the fifth lane is M6PR antibody to Gimap5 IP product of Beas-2b cells; and the sixth lane is M6PR antibody to the Gimap5 IP product of Beas-2b-Gimap5 cells. **(B)** Secondary peptide spectra of M6PR were detected in the Co-IP products using Gimap5 antibody. **(C)** the total protein of Beas-2b was extracted, and the interacting proteins of M6RP were screened using Co-IP. Western blot using anti-Gimap5 antibody showed that M6RP interacted with Gimap5. **(D)** The expression levels of M6PR in Co-IP products of Gimap5 and the expression levels of Gimap5 in Co-IP products of M6PR antibody were detected using Western blot. **(E)** The GO analysis of mass spectrometry of Gimap5 interacted proteins. **(F)** The GO analysis of mass spectrometry of M6PR interacted proteins.

After the successful construction of Gimap5 overexpressed Beas-2b and PC9 cell lines, we screened the interacting proteins of Gimap5 using Co-IP and tandem mass spectrometry with the rabbit anti-Gimap5 antibody (**Figure 5A**). The peptide segment of M6PR was found using tandem mass spectrometry (**Figure 5B**). Furthermore, the total protein of Beas-2b was extracted. The interacting proteins of M6RP were screened using Co-IP and detected with the anti-Gimap5 antibody using a western blot system. Findings showed that M6RP interacted with Gimap5 (**Figure 5C**). The expression levels of M6PR in Co-IP products using the Gimap5 antibody, as well as the expression levels of Gimap5 in Co-IP products using the M6PR antibody, were detected using a western blot system. According to data analysis, no significant differences in the expression levels were observed (**Figure 5D**). The GO analysis showed that Gimap5 promoted the function of anti-infection, cytoplasmic vesicle lumen, secretory granule lumen and lysosome (**Figure 5E**). After rabbit-anti M6PR was added to the immunoprecipitate of Gimap5, the mass spectrometry results indicated that M6RP interacted with protein-arginine deiminase type-4 (PADI4), actin, cytoplasmic 2 (ACTG1), Keratin, type II cytoskeletal 6A (KRT6A), breast cancer anti-estrogen resistance protein 1 (BCAR1), and transcription factor TFIIIB component B homolog (BDP1) (**Figure 5F**).

### Co-Location of Gimap5 and M6PR and Its Influence on M6PR Distribution

**Figure 6:**
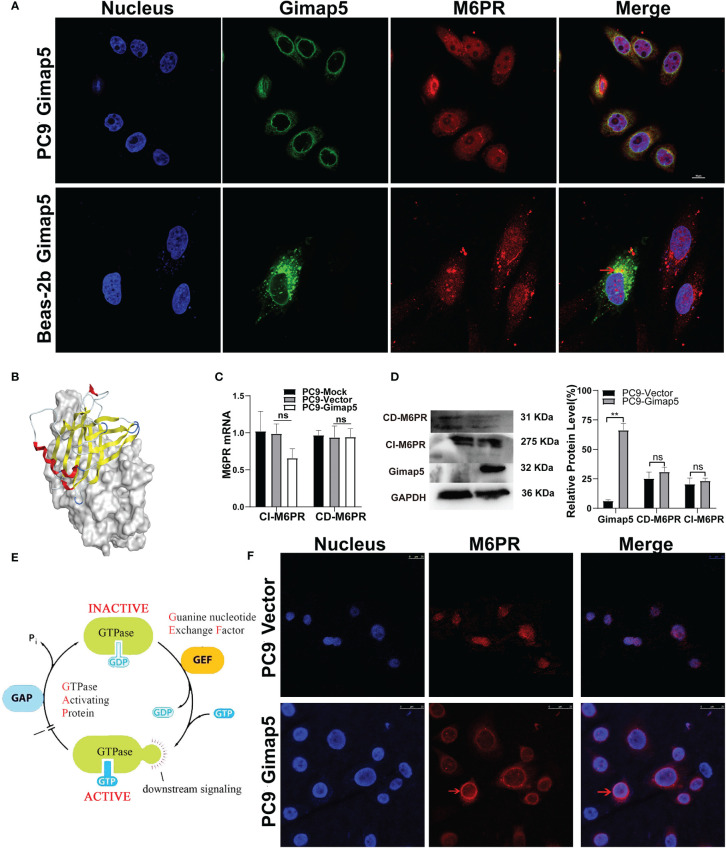
Gimap5 altered the distribution of M6PR on the lung cancer cells surface. **(A)** The co-location of Gimap5 and M6PR were examined in PC9-Gimap5 cells and Beas-2b-Gimap5 cells. (magnification, × 1000). **(B)** Molecular docking diagram of Gimap5 and M6PR. **(C)** The effect of Gimap5 overexpression on the transcription level of M6PR (ns means no significance). **(D)** The effect of Gimap5 overexpression on the protein level of M6PR. After the image scanning gray analysis, the internal reference GAPDH correction was used to obtain the ratio of each group (ns means no significance; **p < 0.01 vs. empty vector). **(E)** Pattern diagram of action mechanism of GIMAP5 GTPase. **(F)** Gimap5 altered the intracellular distribution of M6PR (magnification, × 400).

Using bioinformatics software, we analyzed and predicted the subcellular localization of GIMAP5 and related functional and interacting proteins (**Supplementary Figure 2**). Meanwhile, Western blot assay was used to detect the protein expression level of Gimap5 overexpression (**Supplementary Figure 3**). To verify the interaction between Gimap5 and M6PR, we detected the co-localization of Gimap5 and M6PR in the cells *via* indirect immunofluorescence (**Figure 6A**). The results showed that Gimap5 and M6PR were co-localized in the cytoplasm. We then performed molecular docking of Gimap5 and M6PR (**Figure 6B**). The score of 77 indicated a strong interaction between M6PR and Gimap5. The analysis of the interaction region between Gimap5 and M6PR was shown in **Supplementary Figure 4**. To ascertain how Gimap5 regulated M6PR, we detected the expression levels of M6PR mRNA in PC9 after Gimap5 overexpression and found no significant changes in their mRNA levels (**Figure 6C**). Besides, no significant changes were observed in the protein levels after Gimap5 overexpression (**Figure 6D**). Given that Gimap5 is a kind of GTPase, we speculated that it might play a role by changing the distribution of M6RP (**Figure 6E**). We further observed the distribution changes of M6RP in the cell samples. The results of fluorescence confocal detection showed that after Gimap5 overexpression, the intracellular distribution of M6PR changed significantly, shifting from the cytoplasm to the membrane (**Figure 6F**). This outcome suggested that Gimap5 promoted the transfer of M6PR to the cell membrane and might inhibit the growth of lung cancer cells *via* the processing of M6P modified ligands.

### M6PR Downregulated the Expression of PADI4

**Figure 7:**
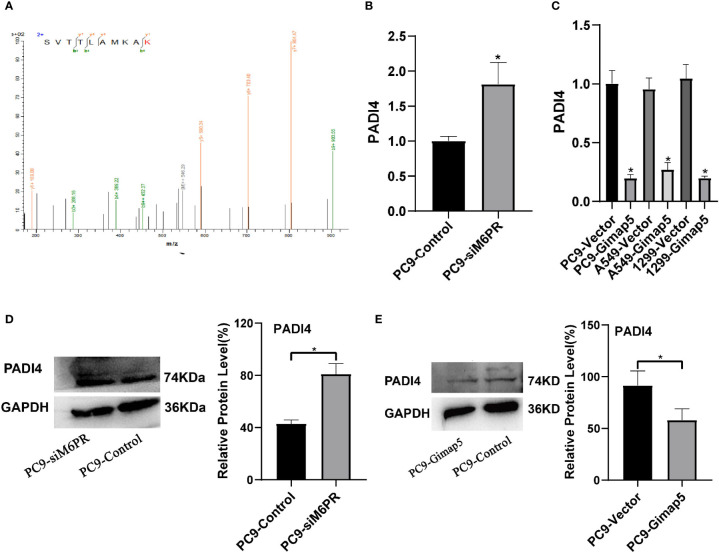
Gimap5 down-regulates the expression of PADI4 (n = 3). **(A)** Secondary peptide spectra of PADI4 were detected in the Co-IP products using M6PR antibody. **(B, D)** the mRNA and protein expression levels of PADI4 after siM6PR transfection was assayed using qRT-PCR and western blot. **(C, E)** the mRNA and protein expression levels of PADI4 after Gimap5 overexpression was detected using qRT-PCR and western blot. All the data were expressed as mean ± SEM of the three independent experiments (*p < 0.05 vs. empty vector).

PADI4 was detected in the IP products of M6PR using tandem mass spectrometry (**Figure 7A**). After detecting si-M6PR in PC9 cells, PADI4 was found to be significantly upregulated (**Figures 7B, D**), while PADI4 was considerably downregulated in cell lines with Gimap5 overexpression (**Figures 7C, E**). As PADI4 is a positive regulator of EMT, we further clarified the role of Gimap5 in inhibiting lung cancer *via* EMT downregulation. At the same time, we detected the expression levels of PADI4 and M6PR in different tumor cell lines, and found that PADI4 increased abnormally in tumor cells, but there was no significant difference in different tumor lines. On the contrary, the expression of CD-M6PR and CI-M6PR was significantly decrease in tumor cells, but there was no significant difference in different tumor cells (**Supplementary Figure 5**).

## Discussion

The invasion and migration of tumor cells lead to the poor prognosis and recurrence of diseases. Studies have shown that the imbalance of GIMAP family expression is related to the development of lung cancer (30, 31). Based on the prognostic value of the GIMAP family in lung cancer, it is possible to understand the mechanism driving the occurrence and development of lung cancer. In this study, we conducted a preliminary study on the mechanism of Gimap5 in lung cancer progression. Our results showed that the expression of Gimap5 was low in lung cancer tissues and cells. Also, this downregulation was found to be related to the poor prognosis of lung cancer. In short, our finding suggested that Gimap5 might be used as a biomarker for the diagnosis and prognosis of lung cancer.

Our follow-up study also showed that the function of Gimap5 depended on its interacting protein M6PR, a tumor suppressor gene. In their study on colorectal cancer cells, Souza et al. reported that the overexpression of M6PR restrained the proliferation of tumor cells (32). The inhibitory ability of M6PR has also been verified *in vivo* for choriocarcinoma and breast tumor cells (25). Previous research suggested that the anti-proliferative activity of M6PR in tumor cells could be traced to its influence on IGF-II signal (23, 33). More specifically, the downregulation of M6PR has been shown to increase the growth rate and tumor incidence of choriocarcinoma cells (34). This result further emphasized the correlation between M6PR status and tumor progression. In one study, it was proven that M6PR could interact with matrix degradation protease to inhibit the tumorigenicity and invasiveness of squamous cell carcinoma cells. Rupal and Tang found that the exposure of M6PR on tumor cells could make GrzB produced by activated cytotoxic T lymphocyte (CTL) to penetrate into tumor cells, thus enhancing the cytotoxicity of CTL (35–37). This enhancement suggested that Gimap5 could be a therapeutic target for lung cancer.

**Figure 8:**
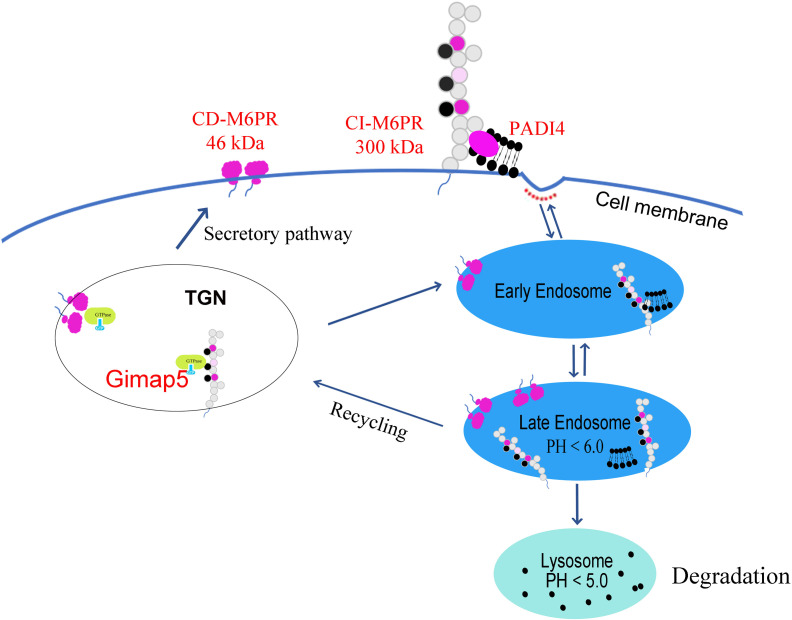
Gimap5 promoted the transport of M6PR from cytoplasm to cell membrane through the interaction with M6PR, and M6PR inhibited the function of M6P modified ligands such as PADI4, thus inhibiting PADI4-related EMT and lung cancer growth.

Furthermore, our study confirmed that the distribution of M6PR shifted from the cytoplasm to the membrane after Gimap5 overexpression, thus promoting the exposure of M6PR. M6PR has been identified to influence the invasion and migration of several types of tumors (38). Therefore, we believed that Gimap5 might play a role in the degradation of M6P modified ligands (i.e., PADI4) *via* M6PR, thereby inhibiting the growth of lung cancer (**Figure 8**). Current studies have revealed the role of GIMAP5 in tumor inhibition. However, the detailed mechanism needs to be further verified by *in vivo* experiments By doing so, it would be possible to provide evidence for the treatment and diagnosis of lung cancer and improve the 5-year survival rate of patients with lung cancer.

All in all, in this study, we found that the expression of Gimap5 in lung cancer cells and tissues decreased considerably and that the low expression of Gimap5 predicted the poor clinical prognosis of lung cancer patients. Findings also revealed that Gimap5 inhibited the invasion, migration, proliferation and EMT of lung cancer cells. Overall, our research suggested that Gimap5 could inhibit the growth of lung cancer by interacting with M6PR and that it could be a potential biomarker for the diagnosis and prognosis of lung cancer.

## Data Availability Statement 

The datasets presented in this study can be found in online repositories. The names of the repository/repositories and accession number(s) can be found in the article/**Supplementary Material**.

## Ethics Statement

Ethical review and approval was not required for the study on human participants in accordance with the local legislation and institutional requirements. Written informed consent for participation was not required for this study in accordance with the national legislation and the institutional requirements.

## Author Contributions

YT designed this work. PD wrote the paper and carried out the experiments. ZT, PR, DL and OB analyzed the data. All authors contributed to the article and approved the submitted version.

## Funding

This work was supported by Grant 31771277 from National Natural Science Foundation of China and Central South University Postgraduate Innovation Project (2021).

## Conflict of Interest

The authors declare that the research was conducted in the absence of any commercial or financial relationships that could be construed as a potential conflict of interest.

## Publisher’s Note

All claims expressed in this article are solely those of the authors and do not necessarily represent those of their affiliated organizations, or those of the publisher, the editors and the reviewers. Any product that may be evaluated in this article, or claim that may be made by its manufacturer, is not guaranteed or endorsed by the publisher.
