# Supplementary material for: Gimap5 Inhibits Lung Cancer Growth by Interacting With M6PR
Source: Front Oncol. 2021 Sep 15;11:699847. doi: 10.3389/fonc.2021.699847 (PMC8479171; doi:10.3389/fonc.2021.699847)
Supplement: Supplementary file 1 [file DataSheet_1.docx]

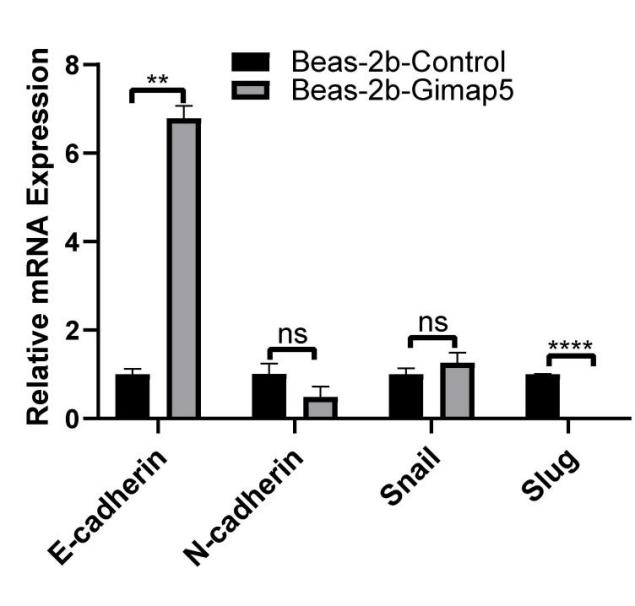


**Supplementary FIGURE 1** Expression of EMT-related factors in Beas-2b cells after overexpression of Gimap5 was assayed using RT-qPCR.

**
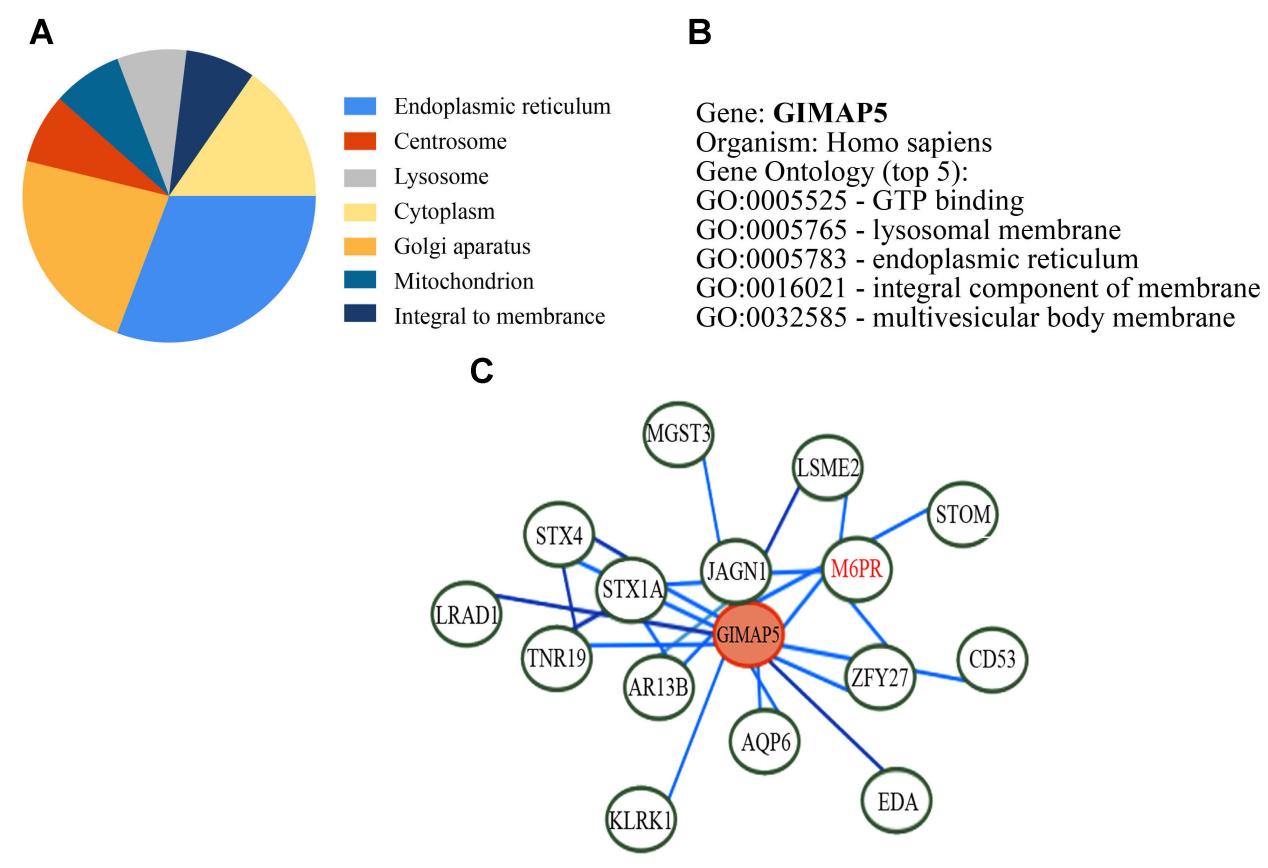
**

**Supplementary FIGURE 2** The function of Gimap5 was analyzed using bioinformatics.

1. Subcellular localization of GIMAP5. (B) Biological function prediction of GIMAP5. (C) Predictions about GIMAP5 interacting proteins were derived from STRING's PPI network data.


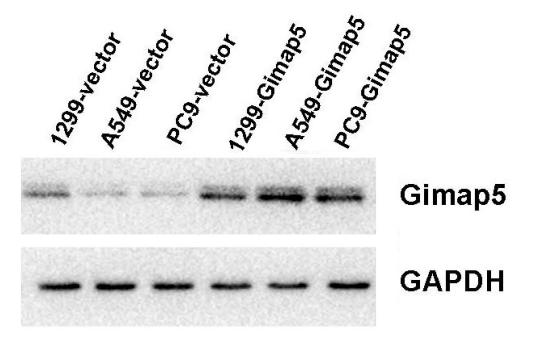


**Supplementary FIGURE 3** Gimap5 was overexpressed in lung cancer cell lines was assayed using Western blot


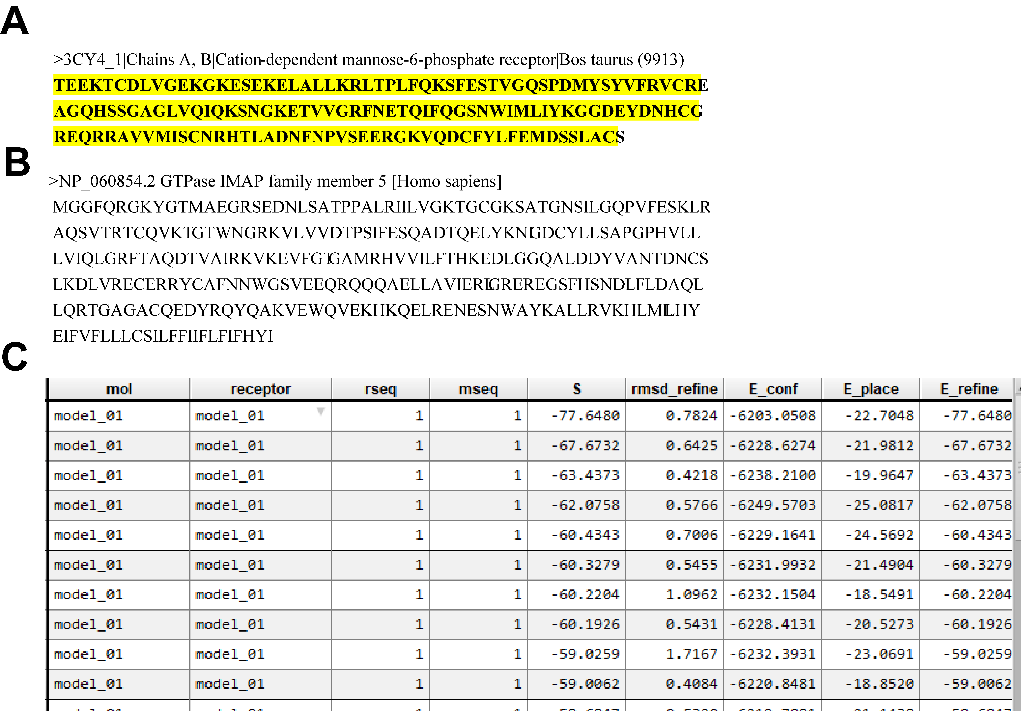


**Supplementary FIGURE 4** The analysis of the interaction region is about Gimap5 and M6PR. (A) Amino acid sequence of GIMAP5 interacting with M6PR. (B) Amino acid sequence of Gimap5. (C) Molecular docking score of Gimap5 and M6PR.


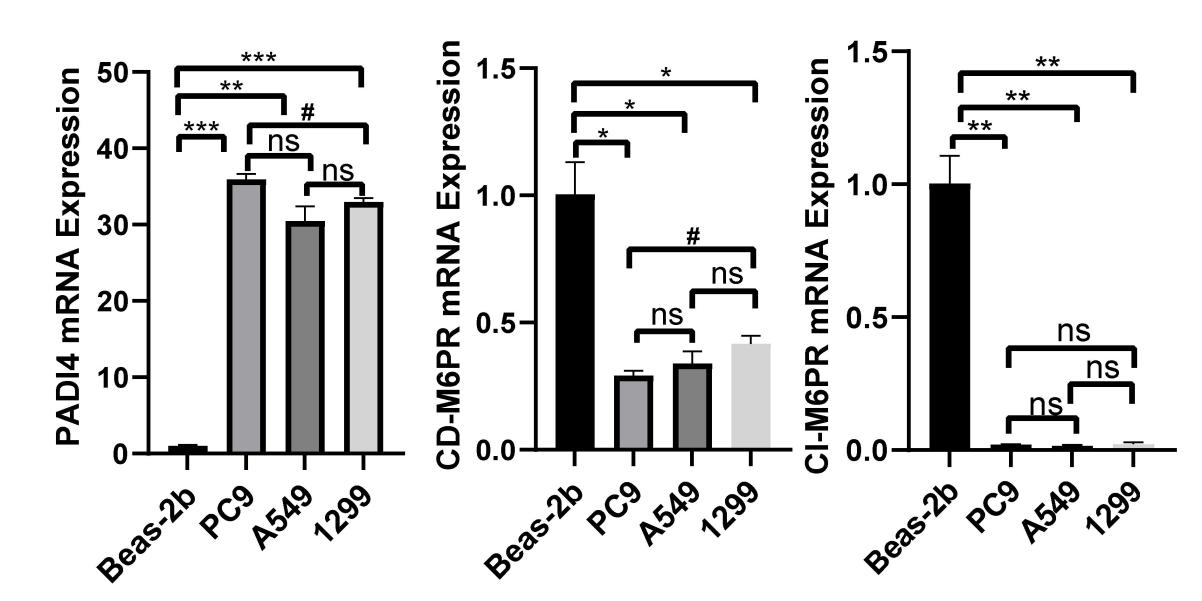


**Supplementary FIGURE 5** Expression of PADI4 and M6PR in different tumor cells.
